# Mycophenolate Mofetil for Systemic Lupus Erythematosus: Our 20-Year Experience

**DOI:** 10.7759/cureus.34413

**Published:** 2023-01-30

**Authors:** Michael Trevisonno, Alexander Hall, Sabrina Rosengarten, Ellen M Ginzler

**Affiliations:** 1 Rheumatology, State University of New York Downstate Health Sciences University, Brooklyn, USA

**Keywords:** long-term outcome, sle and lupus nephritis, mycophenolate mofetil, lupus nephritis, systemic lupus erythromatosus

## Abstract

Objectives: Mycophenolate mofetil (MMF) has been long used in the treatment of systemic lupus erythematosus (SLE). Further studies are warranted to investigate its long-term use in maintenance treatment of lupus nephritis (LN). The purpose of this study was to describe our practice experience using MMF with regard to its indications, safety, tolerability, and treatment efficacy. We sought to identify rates of renal remission, flare and progression to end-stage renal disease (ESRD).

Methods: In this retrospective chart review, we identified all patients treated with MMF between 1999 and 2019. Descriptive statistics were used to identify occurrence of remission, occurrence of flares, progression to ESRD, and occurrence of adverse effects.

Results: One hundred and one patients were treated with MMF for a mean duration of 69 months. The most common indication was LN (90%). Among patients with LN, 60% achieved complete remission and 16% achieved partial remission at one-year follow-up. Ten patients flared while on maintenance therapy and seven patients flared after treatment was discontinued. Of the 40 patients who were treated for at least five years, one patient developed a flare. Of the 13 patients who were treated for at least 10 years, none developed a flare. One patient on maintenance therapy progressed to ESRD. The most common adverse effects were leukopenia (9%), nausea (7%) and diarrhea (6%).

Conclusion: Maintenance treatment with MMF constitutes an effective long-term treatment for lupus nephritis. Our practice demonstrates its tolerability over many years with few adverse effects, prevention of renal flares, and a low progression rate to ESRD.

## Introduction

Mycophenolate mofetil (MMF) was first approved by the FDA in 1995 for the prevention of transplant graft failure [1]. Its anti-inflammatory and immunomodulatory properties subsequently led to its use in multiple rheumatic diseases. The principal mechanism through which the drug exerts its immunosuppressive effects is by interfering with the purine pathway, upon which activated lymphocytes are dependent. MMF is a pro-drug of mycophenolic acid, which inhibits inosine monophosphate (IMP) leading to decreased B-cell and T-cell proliferation, and decreased antibody production [2].

Several randomized controlled trials have demonstrated MMF’s effectiveness at inducing remission and preventing flares in patients with lupus nephritis. Its safety profile has been favorable compared to other established therapies. Ginzler et al. revealed MMF’s effectiveness at inducing remission and a superior safety profile when compared to cyclophosphamide [3]. Other randomized controlled trials, including ALMS and MAINTAIN, have also established its benefit in treatment induction [4,5]. In addition, MMF has also been shown to be effective in other non-renal manifestations of systemic lupus erythematosus (SLE) by reducing steroid requirements and the number of subsequent disease flares [6,7]. 

MMF has now been the standard of care for lupus nephritis for more than 20 years. Despite its long history and documented effectiveness, particularly in the treatment of lupus nephritis, further studies are warranted to investigate its role in long-term maintenance therapy. The purpose of this study was to describe our 20 years of practice experience using MMF with regard to its indications, safety, tolerability and treatment efficacy. Among patients on long-term treatment, we sought to identify rates of renal flare and progression to end-stage renal disease (ESRD).

This article was previously presented as a poster at the 2020 American College of Rheumatology Convergence on November 9, 2020. 

## Materials and methods

We performed a retrospective review of medical records of patients in the State University of New York (SUNY) Downstate SLE registry. We identified all patients with SLE who were treated with MMF between 1999 and 2019. Data were collected on demographics, indication and duration of treatment, reason for discontinuation, and side effects. Among patients with lupus nephritis, data was collected on occurrence of renal remission, flare and progression to ESRD.

Remission and flare definitions were based on those from the MAINTAIN trial [5]. Complete remission was defined as a return to normal serum creatinine (Cr; <1.4) and <0.5 g/day proteinuria. Partial remission was defined as an improvement of 50% in all abnormal renal measurements (Cr and proteinuria) as long as in sub-nephrotic range. Renal flare was defined as the recurrence of nephrotic syndrome or, only for patients with low-grade baseline 24-hour proteinuria (>0.5 and <1 g), a threefold increase of 24-hour proteinuria within a three-month period or renal impairment (>33% increase of serum Cr within a three-month period directly attributed to lupus and confirmed [5].

Descriptive statistics were used to identify occurrence of remission, occurrence of renal flares, and progression to ESRD. A univariate regression analysis was performed to identify predictors of remission and flare.

## Results

Six hundred and sixty patients with SLE were reviewed, of whom 101 were treated with MMF. Patient demographics are listed in Table 1 . Reasons for starting treatment included nephritis (90%), interstitial lung disease (7%), and use as a steroid-sparing agent (4%). The median dose was 3.0 and the mean dose was 2.6 mg/day. Our standard procedure was to not taper the dose of MMF during maintenance therapy unless there were side effects. The mean duration of treatment was 69 months for a total of 580 patient-years. Ten percent of patients were treated for less than one year, 45% for one to five years, 29% for five to 10 years and 17% for over 10 years. Ninety-two patients (92%) had lupus nephritis. Biopsy at presentation showed that 1% had class I, 3% had class II, 8% had class III, 43% had class IV, and 35% had class V.

**Table 1:** Patient demographics and co-morbidities *Values are the number (equivalent to percentage)

Characteristic	Overall population (n=101)
Gender	
Female	85
Male	16
Race	
African American/Caribbean	87
Caucasian	9
Latino	3
Asian American	2
Co-morbidities	
Hypertension	59
Diabetes Mellitus	16
Other rheumatic disease	15
Interstitial lung disease	12
Coronary artery disease	11
Other renal disease	5

Renal remission

After one year of treatment, 60% achieved complete remission, 16% achieved partial remission, and 23% did not achieve remission. The mean serum creatinine was 1.05 mg/dL at baseline, and 0.88 mg/dL after one year of treatment. The mean urine protein/creatinine ratio (UPCR) was 3.01 g at baseline, and 0.67 g after one year of treatment. Among those who did not achieve remission after one year, 50% did within two years. Of those who achieved partial remission after one year, 42% achieved complete remission within two years.

Treatment discontinuation and adverse effects

The reasons for discontinuing treatment included: Non-compliance in nine patients, lack of efficacy in nine, adverse effects in six, pregnancy planning in four and remission in one. Of the six patients whose treatment was discontinued due to adverse effects, thre were due to leukopenia and three were due to gastrointestinal side effects. Table 2 summarizes adverse effects in all patients. Gastrointestinal side effects including nausea, diarrhea and abdominal pain were most common. Among patients with these side effects, seven were continued on MMF as symptoms self-resolved, three were continued on a reduced dose, one was switched to myfortic acid, and three were switched to another medication class.

**Table 2:** Adverse events *Values are the number (equivalent to percentage)

Complications	Overall population (n=101)
Gastrointestinal	
Nausea	7
Diarrhea	6
Abdominal pain	1
Hematology	
Leukopenia	9
Infection	
Zoster	3
Urinary tract infection	2
Pneumonia	2
Candidiasis	2
Other	
Headaches	2
Osteoporosis	2
Lymphoma	1

Renal flare

Ten patients had a renal flare while on MMF and seven patients had a flare after treatment was discontinued. Among patients who flared off treatment, five were non-adherent and self-discontinued treatment, and two had treatment discontinued due to pregnancy. They were all in renal remission, had received treatment for a mean of 41 months, and flared after a mean of 18 months. There were 14 patients who had treatment discontinued for the above-listed reasons who did not experience a renal flare post-treatment. These patients were monitored for a mean of 48 months and a median of 39 months post-treatment without flare. Table 3 and Table 4 summarize the characteristics of patients who flared while on maintenance therapy and after post-discontinuation respectively. Of the 40 patients who were treated for at least five years, one patient (2%) developed a flare. Of the 13 patients who were treated for at least 10 years, none developed a flare. A univariate analysis did not identify any significant predictor of flare, including age, race, gender, class of nephritis, and baseline labs (anti-dsDNA antibody, C3, C4, serum creatinine, and UPCR).

**Table 3:** Characteristics of patients who experienced a renal flare while on maintenance treatment MMF: mycophenolate mofetil, ESRD: end-stage renal disease

Months on MMF	MMF continued after flare	Medication added at time of flare	Return to remission
7	Yes	Rituximab	Complete
16	Yes	Steroids	No -> ESRD
19	Yes	Cyclophosphamide	Complete
22	Yes	Rituximab	Partial
36	Yes	Increased MMF	Partial
37	No	Cyclophosphamide	Complete
39	Yes	Cyclophosphamide	Complete
39	Yes	Steroids	Partial
48	Yes	Steroids	Complete
64	Yes	steroids	Complete

**Table 4:** Characteristics of patients who experienced a renal flare after treatment was discontinued MMF: mycophenolate mofetil, ESRD: end-stage renal disease

Months since discontinuation	Time spent on MMF	Reason for discontinuation	MMF re-started	Return to remission
1	24	Self-discontinued	Yes	Complete
2	81	Self-discontinued	No	No -> ESRD
2	60	Self-discontinued	Yes	Partial
6	36	Self-discontinued	Yes	Partial
15	24	Pregnancy	Yes	Partial
23	50	Pregnancy		No
81	12	Self-discontinued	No	No -> ESRD

ESRD

Five patients progressed to ESRD including one patient who was maintained on MMF, two patients who never achieved remission, and two patients who discontinued use due to non-compliance. The one patient on maintenance treatment flared after 16 months of induction therapy. She did not respond to increased steroid doses and progressed to ESRD. One patient who self-discontinued treatment received MMF for 81 months, then progressed to renal failure after two months of treatment discontinuation. The other patient who self-discontinued treatment took MMF for 12 months, then progressed to renal failure after 81 months of discontinuation.

## Discussion

The treatment of lupus nephritis consists of two phases, an induction phase to induce remission, and a maintenance phase to uphold the response. Induction trials have been easier to perform given their short-term assessment, and they have persuasively shown that MMF is effective at inducing remission [3,4]. Our study further supports this research, with 76% of patients achieving either complete or partial remission after one year. In addition, a large number of patients who did not achieve remission after the first year did so after two years. The majority (87%) of patients in this study were African American/Caribbean. Although this population has been associated with poor outcomes [8], the remission rate in our patient group has been comparable or superior to those in other studies. This successful induction response may be due to our high target dose of 3 g/day.

This retrospective review demonstrates the long-term efficacy of MMF, with few patients flaring on long-term maintenance therapy. Our patients appear to flare less frequently than those in other trials. For instance, in the long-term follow-up of the MAINTAIN trial, 19 of 42 patients experienced a flare within the 10-year follow-up [9]. As mentioned above regarding induction, our reduced flare rate during maintenance treatment might also be attributed in part to dosing. We have maintained our patients on 3 g/day, whereas other trials had lower induction target doses and subsequent dose tapering during maintenance [10]. There is a lack of trials that investigate whether MMF can be safely discontinued in patients with inactive lupus nephritis. Prior studies have suggested that flares may occur when treatment is reduced [10].

We were unable to compare the flare rate in patients who were maintained on treatment to those who were tapered off due to inactivity as there was only one such patient. Our approach was to maintain patients on MMF despite duration of remission. Our low flare rate and low frequency of adverse events support this approach, with only six patients needing to discontinue treatment due to either leukopenia or gastrointestinal side effects. Further supporting the plan for long-term maintenance is the high number of patients who flared after treatment was discontinued. These seven patients stopped treatment due to non-compliance or pregnancy planning; all were in remission and were previously treated for a mean of 41 months. An opposite conclusion was drawn in a retrospective study of 44 patients with the finding that reducing MMF after more than 18 months of therapy was not associated with increased relapse rates [11]. Controlled studies are warranted to compare outcomes of tapering versus maintaining treatment. 

Our study did not identify any predictors of renal flare. This is likely due to the low number of patients that experienced a flare. Other studies have shown that predictors of flare in SLE patients include younger age, male gender, and diffuse proliferative lupus nephritis on renal biopsy [12]. Regarding predictors of response to MMF, one systematic review concluded that there is not enough data for any predictor, and would not recommend any to be considered in clinical practice [13].

## Conclusions

Our study is limited by its retrospective analysis. The variations in dosing, timing, and follow-up intervals are a limitation. Nonetheless, we can conclude that MMF is effective at inducing complete or partial remission and at preventing renal flares. Long-term maintenance therapy is associated with a low flare rate, and long-term use is safe with few adverse effects. In over 20 years of use in patients with SLE, no new safety signals have been recognized.
